# Rapid low dose electron tomography using a direct electron detection camera

**DOI:** 10.1038/srep14516

**Published:** 2015-10-05

**Authors:** Vadim Migunov, Henning Ryll, Xiaodong Zhuge, Martin Simson, Lothar Strüder, K. Joost Batenburg, Lothar Houben, Rafal E. Dunin-Borkowski

**Affiliations:** 1 Ernst Ruska-Centre for Microscopy and Spectroscopy with Electrons and Peter Grünberg Institute, Forschungszentrum Jülich, D-52425 Jülich, Germany; 2 PNSensor GmbH, Otto-Hahn-Ring 6, 81739 München, Germany; 3 Centrum Wiskunde & Informatica, P.O. Box 94079, NL-1090 GB Amsterdam, The Netherlands; 4 PNDetector GmbH, Otto-Hahn-Ring 6, 81739 München, Germany; 5 University of Siegen, Walter Flex Str. 3, 57068 Siegen, Germany; 6 Mathematical Institute, Leiden University, The Netherlands; 7 iMinds-Vision Lab, University of Antwerp, Belgium

## Abstract

We demonstrate the ability to record a tomographic tilt series containing 3487 images in only 3.5 s by using a direct electron detector in a transmission electron microscope. The electron dose is lower by at least one order of magnitude when compared with that used to record a conventional tilt series of fewer than 100 images in 15–60 minutes and the overall signal-to-noise ratio is greater than 4. Our results, which are illustrated for an inorganic nanotube, are important for ultra-low-dose electron tomography of electron-beam-sensitive specimens and real-time dynamic electron tomography of nanoscale objects with sub-ms temporal resolution.

Electron tomography (ET) is an essential technique for studying the morphology, microstructure and composition of nanoscale materials in three dimensions in the transmission electron microscope (TEM)^1^. It requires the acquisition of a series of images of a specimen in different viewing directions with the highest possible object tilt range and the greatest possible number of projections^2^. The tilt range and tilt increment have a direct impact on the resolution of the final three-dimensional reconstruction^1^. However, when studying specimens that are electron-beam-sensitive or change as a function of time (*e.g.*, catalytic processes and defect nucleation and growth), there is a restriction on the number of projections that can be acquired, especially for applications of ET in the life sciences^3^ and for studies of soft matter (*e.g.*, polymers)^4^,^5^. The development of rapid image acquisition is therefore of great importance for both low dose ET and time-resolved three-dimensional studies of dynamic processes, such as phase transformations and switching mechanisms^6^,^7^.

Continuous image acquisition in the medical sciences using X-ray computed tomography (CT)^8^, involving rotation of a scanner and translation of a patient, has provided sub-second tomographic acquisition since the late 1990 s^9^, with the required hardware development facilitated by the possibility of rotating both the source and the detector independently and by the almost complete absence of restrictions in available space. In electron microscopy, similar hardware developments are extremely difficult to achieve and the fast and efficient detection of electrons has been an obstacle. An increase in the speed of tomographic acquisition has only been gained by the automation of tilt series acquisition and alignment^10^, with the recording of a tilt series of images still typically taking several tens of minutes.

Here, we demonstrate rapid TEM tomography by making use of recent advances in direct electron detection cameras^11^,^12^,^13^,^14^, whose high detective quantum efficiency (DQE) and fast read out provide improved spatial resolution and detectability^15^. We acquire a tomographic tilt series of images while continuously rotating the microscope goniometer, resulting in a frame rate of approximately 1000 fps. We demonstrate the ability to record a tilt series of images in only a few seconds, *i.e.*, approximately a hundred times faster than in conventional ET, thereby reducing the total electron dose by approximately one order of magnitude when compared to typical low dose ET in the life sciences.

In order to demonstrate rapid tomographic image acquisition, we studied an individual lanthanide-based inorganic nanotube on a lacey C support film. Such nanotubes are an example of a new class of nanotubular structures^16^ that are formed by folding misfit layered compounds, which comprise alternating crystallographic layers with different periodicities. In the present sample, LaS layers that have a rock-salt structure and are alloyed with Ce alternate with layers of hexagonal CrS_2_. Tomographic tilt series were recorded around a single rotation axis in an FEI Titan 60–300 TEM operated at 60 kV. A direct electron detection camera equipped with a pnCCD sensor^17^, based on a similar setup used previously for fast spectroscopic X-ray imaging^18^, was mounted below the projection chamber and used to record bright-field TEM images. The sensor was operated in averaging mode at a frame rate of more than 1000 fps.

Figure 1 shows individual images taken from a series of 3487 frames recorded during rotation of the goniometer over a tilt range of −70° to +30° under continuous electron beam illumination. The complete tilt series can be seen in Supplementary Movie M1. Continuous rotation and recording eliminated the need for low-dose techniques. The tilt range was limited only by the precision with which the mechanical rotation of the goniometer was able to keep the region of interest within the field of view and in focus. The high DQE of above 0.9 and the low dark noise of approximately 0.4% with the sensor running in averaging mode allowed the tilt series to be acquired with a total of ~1.34 × 10^5^ electrons in each frame, corresponding to ~2 electrons per pixel per frame. The cumulative dose for the sum of all of the frames was ~8 electrons per Å at a signal-to-noise ratio of ~2. Prior to reconstruction, the images were aligned to compensate for lateral displacements between successive projections resulting from mechanical sample tilt. Sub-pixel alignment was achieved by using an iterative feedback algorithm that optimizes the tomogram contrast and resolution^19^. The alignment was carried out on a reduced dataset consisting of a running average of five neighbouring projections and interpolated to obtain the displacements for all 3487 frames.

Tomographic reconstruction was performed on the reduced dataset, since its signal-to-noise ratio is twice as high (4.4 instead of 2.2), using both the standard simultaneous iterative reconstruction technique (SIRT)^20^ and the discrete algebraic reconstruction technique (DART)^21^,^22^. A traditional algorithm such as SIRT requires a large number of projections and suffers from missing wedge artifacts when the tilt range is limited. In contrast, DART reduces the amount of information required for reconstruction based on prior knowledge that the material composition can be segmented into a few classes and provides an improved reconstruction with significantly reduced missing wedge artifacts. The reconstructed three-dimensional morphology of the nanotube is shown in Figs 2 and 3 and in Supplementary Movie M2. The SIRT and DART reconstructions show the nanotube clearly, *i.e.*, a torus in cross-section (the *yz* plane in Fig. 3) and the surfaces of the inner and outer walls in axial sections (the *xy* and *yz* planes). The SIRT reconstruction is elongated in cross-section (in the *yz* plane) and contains artefacts in the *z* direction due to the “missing wedge”. The DART reconstruction suffers from noise in the original dataset, giving rise to artificial roughness of the inner walls of the nanotube (in the *xy* and *xz* planes), which is smoothed in the case of SIRT. Both techniques provide reconstructions that can be used for further analysis of the three-dimensional morphology of the nanotube.

The tomographic reconstruction results shown above demonstrate that rapid acquisition of a tilt series is facilitated by the use of a fast direct electron detector, providing improved dose efficiency and time resolution in ET. Considering the significant oversampling in angular tilt space in our experiment, a further reduction in recording time is possible for a full tomographic tilt series. The present limitation is the speed and control of eucentricity of the stage tilt in a mechanical electron microscope goniometer. It currently takes ~5.6 s to tilt the specimen over ±80°. Both limitations can be overcome by introducing fast, precise, highly eucentric specimen stages. Based on the present experiments, we estimate that rotating the specimen stage 10 times faster will not introduce motion blurring in individual micrographs (see the Supplementary Information for further details). Image acqusition at 1000 frames per second could then allow a ±80° tilt series to be acquired in 0.6 s with a total of ~600 images (instead of the ~3500 used here). Such settings will also provide significantly improved lateral resolution due to the decreased tilt increment (~ 0.3°) when compared to conventional ET, which uses tilt increments of 1-2°. Ultimately, a full tilt range of ±90° in such experiments should be achievable^23^.

The rapid acquisition of tomographic tilt series opens new horizons for performing ET of ultra-beam-sensitive specimens in the life sciences or in studies of soft matter, where the total electron dose is of primary importance. We were able to reduce the electron dose by at least an order of magnitude when compared to that used in conventional cryo-electron tomography^24^. Fast tomography can also be combined with existing TEM tomography techniques that makes use of energy filtering for contrast enhancement and chemical sensitivity, as well as phase contrast techniques and cryo-tomography of non-stained samples. Electronic focus and displacement compensation for the imperfect motion of a mechanical goniometer can be calibrated from a prior tracking run performed just outside the field of view. The technique can also be combined with real-time three-dimensional reconstruction. The present dataset could be reconstructed using a reduced subset in a few seconds (see the Supplementary Information for further details). Including data acquisition, an initial reconstructed volume could be obtained in less than 15 s using standard computing power.

Continuous rotation of the specimen stage offers a potential breakthrough for studies of dynamic processes *in situ* in the TEM^7^. A temporal resolution of a few seconds or better can be combined with sub-nm spatial resolution in all three dimensions, providing a capability similar to that reported recently for X-ray microtomography of living cell evolution (where each tomographic series was acquired in 18 s)^25^, but with a spatial resolution that is 2–3 orders of magnitude better. Such dynamic tomography can also be combined with energy-filtered TEM^1^ for studies of changes in chemical composition, with off-axis electron holography for dynamic studies of electric and magnetic fields in three dimensions and with the application of stimuli to the sample (*e.g.*, mechanical forces, electrical biasing, applied magnetic fields or a gas environment). The combination of continuous rotation tomography with particle-tracking techniques can also potentially improve temporal resolution to the ms range.

In conclusion, we have demonstrated that, by using currently available TEM hardware and a direct electron detection camera, an electron tomographic tilt series can be acquired in 3.5 s, reducing the total acquisition time for a tilt series by several orders of magnitude and the total electron dose by more than a factor of 10. This capability facilitates ultra-low-dose three-dimensional imaging of beam-sensitive specimens, espeically in the life sciences and in studies of soft matter. It also offers prospects for four-dimensional imaging of dynamic processes *in situ* in the TEM. Further instrumentation development (especially in specimen stages) is required to improve the applicability and robustness of the method.

## Methods

Inorganic lanthanide misfit nanotubes were prepared according to a procedure described previously^16^. For TEM studies, the nanotubes were dispersed onto lacey C grids. Tomographic tilt series were acquired in an FEI Titan 60–300 TEM operated at 60 kV using a Fischione Model 2010 single-tilt tomography holder. A direct electron detection camera with a pnCCD sensor from PNDetector GmbH was mounted below the projection chamber of the TEM. The camera is based on a similar setup used for fast spectroscopic X-ray imaging^18^. The sensor had an imaging area of 12.7 × 12.7 mm, with 264 × 264 pixels that each had a physical size of 48 × 48 μm. The detector is fully depleted and therefore sensitive over its full 450 μm thickness. The sensor is backside illuminated through an unstructured thin entrance window that enables imaging of electrons with energies between 5 and 300 keV. In the present experiment, the detector was adjusted for maximum pixel full well capacity, while still preserving the absolute intensity information. The full well capacity per pixel and per readout cycle is ~24 electrons with an energy of 60 keV each. The full image area was read out at a rate of 1150 frames per second. All images were transferred to the camera control computer and stored on a hard drive for later analysis. No hardware or software link was used to trigger or synchronize acquisition between the TEM and the camera for this measurement. Instead, data acquisition was started and continuous tilt of the sample stage was performed. The precision of the standard goniometer on our microscope allowed the sample to be tilted over 100° with a lateral displacement of the region of interest of no more than ~500 nm, which was adequate for the present study. The change in specimen height over this tilt range was not measured quantitatively but is estimated to be no more than a few tens of nm. In order to reduce dark current noise, the detector was cooled to −15 °C using thermoelectric coolers. A Ti mask was used to transfer heat from the CCD and its carrier board to the coolers. This mask also shielded the edges of the image area and is therefore visible as a dark border in each image. Prior to image acquisition, the specimen was placed at a eucentric height and rotated to −70 °. After acquisition was started, the specimen was tilted to +30°. The asymmetric tilt range is therefore only a consequence of the region of interest no longer being within the field of view above a specimen tilt angle of +30°. The total acquisition time was set to 8 s, whereas the tilt series took only ~3.5 s. The dataset was therefore reduced to those frames during which the sample was rotating (~3500 frames).

## Additional Information

**How to cite this article**: Migunov, V. *et al.* Rapid low dose electron tomography using a direct electron detection camera. *Sci. Rep.* **5**, 14516; doi: 10.1038/srep14516 (2015).

## Supplementary Material

Supplementary Information

Supplementary Movie M1

Supplementary Movie M2

## Figures and Tables

**Figure 1:**
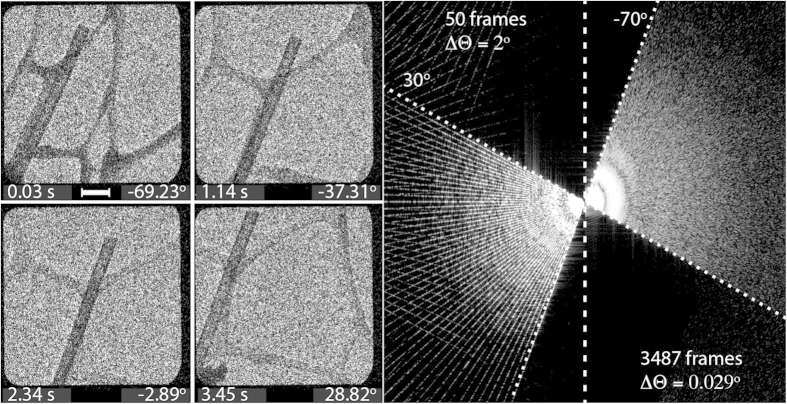
Continuous tilt series acquisition of a (LaCeS)_1.2_CrS_2_ nanotube on a lacey C support. The tilt series comprises 3487 images taken over a tilt range of −70° to +30°. The label in each of the four frames in the left panel indicates the time elapsed and the viewing angle. The scale bar is 200 nm. The right panel shows the filling of Fourier space with projection data according to the Fourier slice theorem, both for a subset of 50 images extracted in 2° increments and for the full tilt series of 3487 images with an increment of ~0.029°. The subset is representative of a conventional tilt series experiment that leaves gaps in Fourier space between successive tilt angles. No gaps are visible for the more densely sampled dataset up to the Nyquist frequency for an image frame size of below 2/ΔΘ ≈ 4 k pixels.

**Figure 2:**
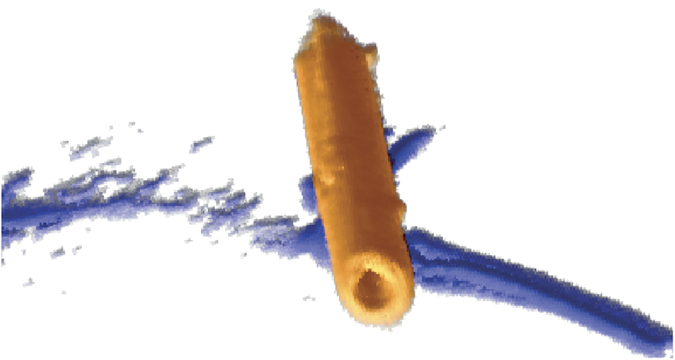
Rendering of a three-dimensional DART reconstruction of the nanotube (orange) and the underlying amorphous C support film (blue).

**Figure 3:**
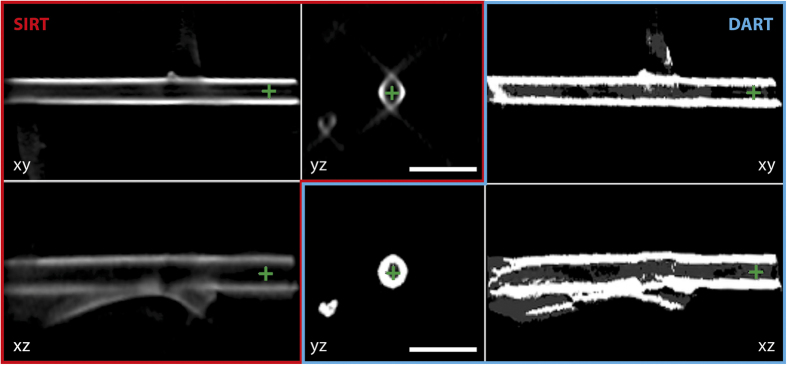
Comparison between SIRT (red frames, left) and DART (blue frames, right) tomographic reconstructions of the nanotube. The centre of reconstructed volume is shown as sections of the *xy*, *yz* and *xz* planes. The green crosses show the positions of the ortho-slices.
