# Supplementary material for: Rapid low dose electron tomography using a direct electron detection camera
Source: Sci Rep. 2015 Oct 5;5:14516. doi: 10.1038/srep14516 (PMC4592966; doi:10.1038/srep14516)
Supplement: Supplementary Information [file srep14516-s1.pdf]

Supplementary Information

Rapid low dose electron tomography using a direct electron detection camera

Vadim Migunov[1*], Henning Ryll[2], Xiaodong Zhuge[3], Martin Simson[4], Lothar Strüder[2,5], K. Joost Batenburg[3,6,7], Lothar Houben[1], Rafal E. Dunin-Borkowski[1]

[1] Ernst Ruska-Centre for Microscopy and Spectroscopy with Electrons and Peter Grünberg Institute, Forschungszentrum Jülich, D-52425 Jülich, Germany

[2] PNSensor GmbH, Otto-Hahn-Ring 6, 81739 München, Germany

[3] Centrum Wiskunde & Informatica, P.O. Box 94079, NL-1090GB Amsterdam, The Netherlands

[4] PNDetector GmbH, Otto-Hahn-Ring 6, 81739 München, Germany

[5] University of Siegen, Walter Flex Str. 3, 57068 Siegen, Germany

[6] Mathematical Institute, Leiden University, The Netherlands

[7] iMinds-Vision Lab, University of Antwerp, Belgium

[*] v.migunov@fz-juelich.de

On the prospect of real-time tomographic reconstruction from tilt series

The ability to record a full tilt series of electron microscope images within seconds is a pivotal requirement for real-time three-dimensional imaging in electron microscopy. Tomographic reconstruction within seconds is facilitated by today's computing power and memory, in combination with the implementation of fast image processing and reconstruction algorithms. In the past, a full tilt-series tomography experiment at high spatial resolution took 20 minutes or longer. As we demonstrate, by using fast tomography one can acquire individual images in a few ms or less. A thousand of these images can be acquired during specimen rotation and combined to reconstruct a full three-dimensional dataset in a few seconds.
Computer control, automated image recording optimised for low-dose acquisition [S1] and the availability of slow-scan CCD cameras have provided a breakthrough for electron tomography, in particular in the life sciences. However, the timeframe for preliminary on-the spot reconstruction has been limited by the cumulative frame recording time of slow-scan CCD cameras and by corrections to the optical system and mechanical goniometer stage. These constraints have limited the ability to obtain preliminary three-dimensional object views to dozens of minutes.
In order to demonstrate the viability of real-time tomographic reconstruction in seconds, we took a subset of the original tilt series of images for automated image handling and weighted backprojection. 100 images were taken from the full tilt series of 3487 images for automated pre-processing and reconstruction. In analogy to Fig. 3 in the manuscript, Fig. S1 shows cross-sectional slices of a tomogram obtained after automated reconstruction in a total processing time of less than 10 s. All essential features of the inorganic nanotube are revealed. Increased speckle contrast when compared to the SIRT and DART reconstruction shown in Fig. 3 results partly from the lower dose associated with the subset selection. The image pre-processing consisted of the following steps: frame cut, contrast inversion, frame padding, frame (tilt axis) rotation, frame (tilt axis) centering, frame cut and pixel-accurate cross-correlation alignment. Table S1 shows a detailed summary of the operation time on a regular Intel 2.26 GHz Core2Duo desktop computer. The pre-processing steps were performed using custom software written in IDL [S2]. Fast weighted backprojection relied on

the multithreaded and vectorized tomo3D code of Agulleiro et al. [S3, S4]. Larger preview reconstructions can be obtained in the same way within seconds by making use of high-performance computing. In particular, the time-consuming pre-processing image rotation and cross-correlation steps are faster on GPUs or accelerator cards.
In conclusion, recording and data processing do not present obstacles for real-time three-dimensional imaging. Limitations currently result from the imperfection of the mechanical goniometer. Future progress would benefit from the use of large pixel array direct electron detectors, together with predictive automation and electronic correction of object displacement.

[S1] A.J. Koster, M. Bàrcena, Cryotomography: Low-dose Automated Tomography of Frozen-hydrated Specimens. In Electron Tomography of Cells and Tissue, 2nd Edition. J. Frank (ed.) (pp. 113- 161). New York: Springer.
[S2] IDL scientific programming language. Exelis Visual Information Solutions. http://www.exelisvis.com.
[S3] J.I. Agulleiro, E.M. Garzon, I. Garcia, J.J. Fernandez, J. Struct. Biol. 170:570-575, 2010.
[S4] J.I. Agulleiro, J.J. Fernandez, Bioinformatics 27:582–583, 2011.

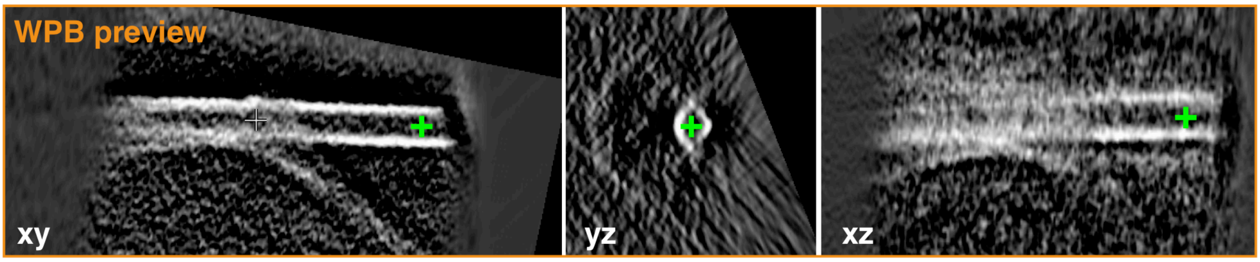

Figure S1. Orthoslices of a preview reconstruction obtained after automated image processing in less than 10 seconds on a dual-core CPU. The weighted backprojection was obtained from a subset of 100 264-pixel images. The sections correspond to those obtained using SIRT and DART reconstructions in Fig. 3 in the manuscript. The green marker indicates a common point in the three-dimensional tomogram of the inorganic nanotube.

Table S1. Processing time for a preview reconstruction on a 2.26 GHz Intel Core2Duo CPU for the reconstruction of a total of 100 264-pixel images. The table is split into automated pre-processing for preparation of the frames for tomographic reconstruction and weighted backprojection on a dual core CPU.

Pre-processing (IDL)

operation	time (s)
cut	0.16
contrast inversion	0.20
padding	1.38
rotation	2.53
centering	0.09
cut	0.11
cross-correlation	2.35
I/O	0.29
total	7.1

Weighted backprojection (tomo3D)

operation	time (s)
reconstruction	0.79
I/O	0.36
total	1.15
